# Reduced Graphene Oxide and Nickel Nanoparticle Nanocomposites: Tailoring the Nanoparticle Size for Enhanced Glucose Electrochemical Sensors

**DOI:** 10.1021/acsomega.6c01043

**Published:** 2026-05-04

**Authors:** Anna E. Silva, Lízia A. X. Bulin, Jean C. Bassani, Drochss Valencia, Daniela Z. Mezalira, Eduardo G. C. Neiva

**Affiliations:** † Departamento de Química, Universidade Regional de Blumenau (FURB), Blumenau, Santa Catarina CEP 89012900, Brazil; ‡ Departamento de Química, Universidade Federal de Santa Catarina (UFSC), Florianópolis, Santa Catarina CEP 88040900, Brazil; § Escuela de Química, 28014 Universidad Industrial de Santander, Bucaramanga, Santander Z.P. 680002, Colombia; ∥ Servicio Geológico Colombiano, Dirección de Laboratorios, Z.P. 111321 Bogotá, Colombia

## Abstract

In this study, we developed an electrochemical glucose sensor using Ni nanoparticles supported on reduced graphene oxide (rGO) with or without polyvinylpyrrolidone (PVP) as a stabilizer. The rGO/Ni nanocomposites were synthesized via a modified polyol method, producing metallic Ni with a face-centered cubic structure. By adjusting the reducing agent ratio, it was possible to control the nanoparticle size and distribution, achieving an average diameter of 4.0 ± 0.9 nm for a Ni^2+^:NaBH_4_ molar ratio of 1:12 (rGO/Ni-4). The electrochemical behavior of the rGO/Ni nanocomposite was evaluated using cyclic voltammetry and chronoamperometry, demonstrating significant activity for glucose oxidation in alkaline media. The sensitivities of the nanocomposite thin films deposited on indium tin oxide substrates were directly influenced by Ni nanoparticle size and by the presence of PVP. The rGO/Ni/PVP-4 nanocomposite exhibited the highest sensitivity (48.6 ± 2.8 μA (mmol L^–1^)^−1^), reaching a very low limit of detection of 0.26 μmol L^–1^. In addition, the optimized Ni-based sensor showed electroactivity across a wide range of glucose concentrations (1–1000 μmol L^–1^) and good reproducibility, although its reuse was limited due to partial loss of the active material. The combination of PVP-assisted stabilization of the nanoparticles and the high conductivity of rGO led to a synergistic improvement in surface area, redox cycling, and electron transfer. These properties make the rGO/Ni/PVP-4 nanocomposite a promising candidate for low-cost, disposable electrochemical sensors for glucose monitoring in biomedical or environmental applications.

## Graphical abstract

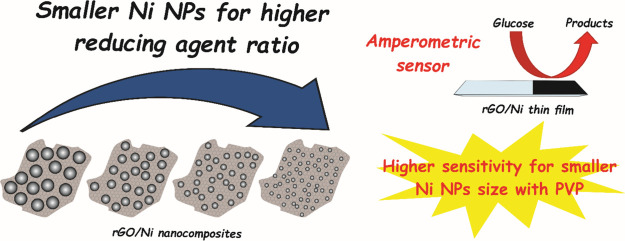

## 1 Introduction

The search for effective glucose monitoring plays a key role in advancing biotechnology, clinical diagnostics, and the food industry.^1^ The urgent need for accurate and affordable glucose determination has led to intensive research efforts aimed at refining analytical methods for glucose detection.^2^ Electrochemical techniques have proven to be highly effective in this field due to their cost-effectiveness, ease of sample preparation, potential for miniaturization, and impressive stability. They also have the advantage of being suitable for full automation.^3^

Traditionally, enzyme-based sensors, such as those using glucose oxidase (GOD), have set the standard for sensitivity and specificity in glucose detection.^4^ However, the high cost of enzymes and their susceptibility to structural degradation during the immobilization process have limited their practical application.^5^ Due to these challenges, the research focus has shifted to nonenzymatic sensing platforms, which offer the dual benefit of cost savings and improved operational stability by eliminating the need for complex enzyme immobilization procedures.^6^ These novel sensors utilize the intrinsic electrocatalytic properties of metallic materials, for example, and simplify the detection process while ensuring high sensitivity, fast response times, and good selectivity^7^ Among the various materials investigated for nonenzymatic glucose sensing, nickel-based catalysts have attracted considerable attention due to their high catalytic activity toward glucose oxidation, good stability in alkaline media, and relatively low cost.^8,9^ Their catalytic performance is mainly associated with the formation of highly oxidative NiOOH species under alkaline conditions, which play a key role in the electrochemical oxidation of glucose.^10^ In this context, it is important to note that both Ni­(OH)_2_ and NiOOH possess two crystalline structures (α- and β-Ni­(OH)_2_, and γ- and β-NiOOH), among which α-Ni­(OH)_2_ and γ-NiOOH are considered the most promising for electrochemical applications due to their higher interlayer spacing and greater structural disorder, which facilitate access to electroactive sites.^11^

Despite the promising advances in nickel hydroxide sensors, there are still limitations regarding material performance.^12^ A promising alternative to overcome these challenges involves integrating metallic catalysts at the nanoscale with nanostructured carbon-based substrates.^11,13^ Graphene, a two-dimensional carbon material, has proven to be an attractive candidate for this purpose due to its excellent electrical conductivity, mechanical strength, and large surface area.^8,14^ These properties make graphene an optimal support for metal nanoparticles in electrochemical applications, enabling efficient electron transfer and improved sensor stability.^15^ Graphene-based materials have been widely investigated as substrates for nickel nanoparticles in glucose sensing applications, enhancing their electrocatalytic activity and long-term stability.^8,16^ The unique properties of graphene, including its ability to facilitate electron transfer and its large surface area, make it an ideal material for improving the catalytic performance of nickel-based systems.

Our study focuses on the synthesis and characterization of rGO/Ni nanocomposites for nonenzymatic glucose sensing. The nanocomposites were synthesized using a modified polyol process that enables precise control over the size and distribution of nickel nanoparticles. The introduction of polyvinylpyrrolidone (PVP) during synthesis and its effect on the size and distribution of Ni nanoparticles were also evaluated. The nanocomposites were structurally, morphologically, and spectroscopically characterized using various techniques. Finally, the electrochemical behavior of rGO/Ni nanocomposites and their electroactivity for glucose detection in alkaline medium were assessed and compared to conventional materials. The combination of nickel nanoparticles and rGO resulted in a synergistic effect: the high conductivity of graphene enabled efficient electron transfer, while the nickel nanoparticles provided active sites for glucose oxidation. Altogether, the rGO/Ni nanocomposites developed in this study represent a promising platform for the development of nonenzymatic glucose sensors. Their superior electrocatalytic activity, combined with the stability and affordability of the materials, makes them strong candidates for practical, high-performance glucose monitoring devices.

## 2 Results and Discussion

### 2.1 Structural and Morphological Characterizations

The structural features of the synthesized rGO/Ni nanocomposites were analyzed by X-ray diffraction (XRD), as shown in Figure 1A. All samples exhibit characteristic diffraction peaks at 44.5°, 51.8°, and 76.5°, corresponding to the (111), (200), and (220) planes of face-centered cubic (fcc) metallic nickel (JCPDS No. 04-0850), confirming the successful reduction of Ni^2+^ to Ni^0^. In particular, an increase in the NaBH_4_/Ni^2+^ molar ratio led to a progressive broadening of these peaks, which is accompanied by a decrease in crystallite size. This decrease in the average crystallite size reflects the effect of faster nucleation and limited growth at high reducing agent concentrations. This behavior is consistent with the classical nucleation theory, that higher supersaturation promotes the formation of numerous small nuclei. As previously reported by the authors,^17^ NaBH_4_ acts as a strong reducing agent that rapidly converts Ni^2+^ to Ni^0^, and increases the nucleation rate. In addition, the rGO/Ni/PVP-4 nanocomposite exhibited the broadest peaks, highlighting the role of PVP as an effective steric stabilizer that inhibits particle growth and aggregation. A weak reflection at 60.7° (*d* = 0.1526 nm), corresponding to NiO, was also detected in this sample. This indicates a partial surface oxidation of the Ni^0^ nanoparticles, probably due to environmental exposure during handling.

**Figure 1:**
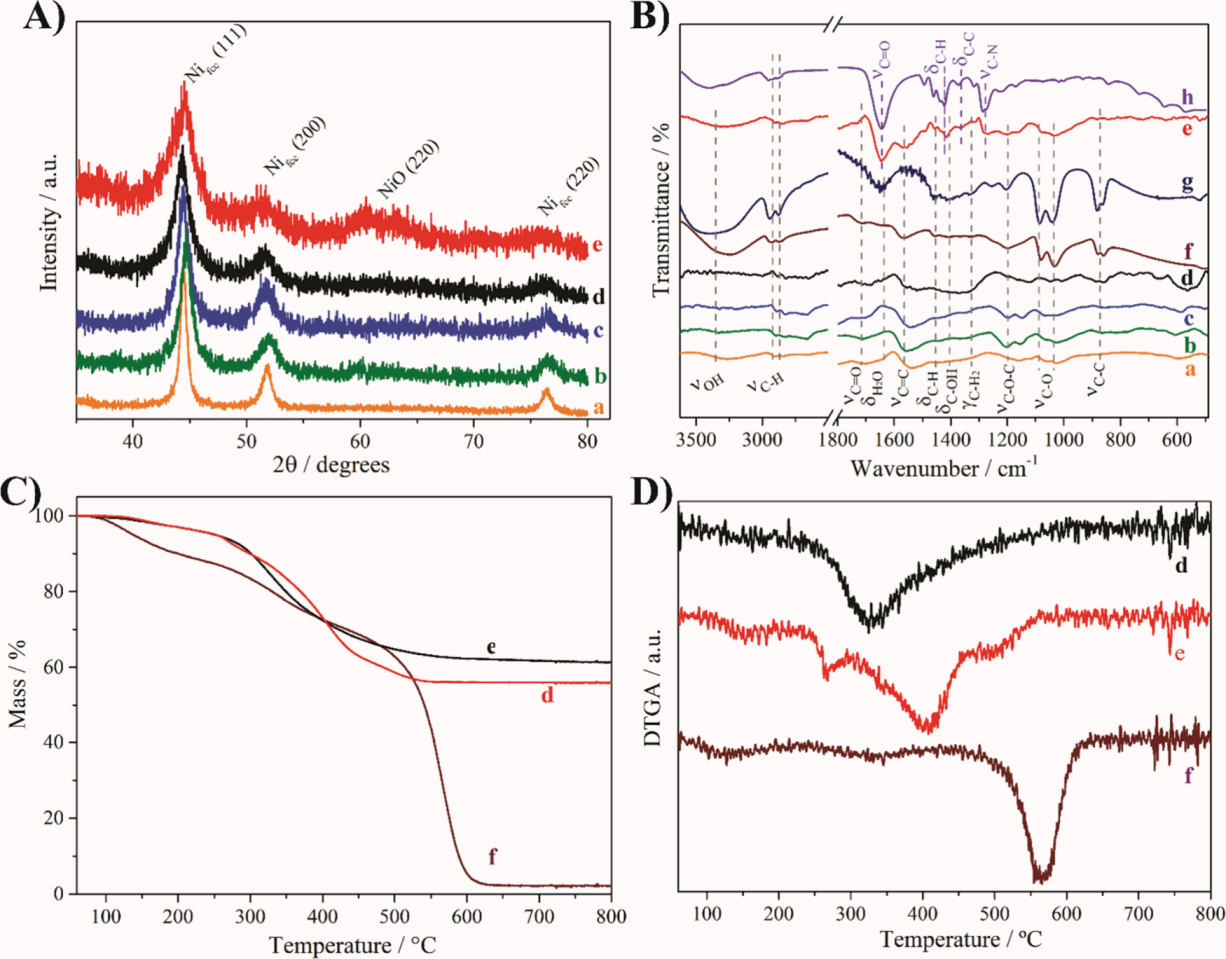
(A) X-ray diffractograms, (B) FTIR spectra, (C) TGA, and (D) DTGA curves of rGO/Ni-1 (a), rGO/Ni-2 (b), rGO/Ni-3 (c), rGO/Ni-4 (d), rGO/Ni/PVP-4 (e), rGO (f), EG (g), and PVP (h). The TGA analyses were carried out under air atmosphere at 10 °C min^–1^, and the samples were first heated at 100 °C for 20 min.

FTIR spectra of the nanocomposites, as well as for rGO, PVP, and EG, are shown in Figure 1B. The rGO/Ni nanocomposites without PVP exhibited bands at 3324/3245 (ν_O–H_), 2929/2853 (ν_C–H_), 1710 (ν_C=O_), 1647 (δ_H2O_), 1563 (ν_C=C_), 1451 (δ_C–H_), 1410 (δ_C–OH_), 1327 (γ_C–H2_), 1200 (ν_C–O–C_), 1080/1032 (ν_C–O_), and 878/860 (ν_C–C_) cm^–1^. These bands are attributed to both residual EG and remaining oxygenated functional groups on rGO such as hydroxyl, carboxyl, carbonyl, and epoxy groups.^17,18^ Their presence indicates that the chemical reduction of GO with NaBH_4_ was partial, which may be beneficial for colloidal stability and electrochemical performance, as these groups may serve as anchoring or electron-mediating sites in redox reactions. The rGO/Ni/PVP-4 nanocomposite showed additional intense bands at 2895/2840 (ν_C–H_), 1641 (ν_C=O_), 1417 (δ_C–H_), 1366 (δ_C–C_), and 1274 cm^–1^ (ν_C–N_) attributed to PVP. It is noteworthy that the band at 1641 cm^–1^ may also contain contributions from EG and/or adsorbed water, indicating possible overlapping vibrations. Comparison of the spectra also shows an increase in the relative intensity of the C–N and C=O signals in the PVP-containing sample, confirming the successful incorporation of PVP. The coexistence of PVP and oxygen-containing groups on rGO likely improves the dispersion of Ni nanoparticles and contributes to a more stable nanocomposite structure, which is consistent with the observed improvements in sensitivity and electrochemical behavior.

Figure 1C shows the TGA curves of the nanocomposites and rGO. The rGO blank presents three distinct mass loss events: (i) from 77 to 187 °C; (ii) from 198 to 384 °C; and (iii) from 415 to 636 °C. The first event can be related to the elimination of EG as well as remaining functional groups on the rGO such as carboxyl and hydroxyl groups. Similarly, the second event is associated with the elimination of remaining functional groups on the rGO such as carbonyl and epoxy groups. The third event is attributed to the oxidation of the carbon skeleton of rGO.^18^ These results confirm the partial reduction of GO, which is consistent with the FTIR spectra, and suggest the presence of thermally labile surface functionalities that may influence the electrochemical behavior of the sensor. The rGO/Ni-4 sample exhibits only two mass loss events. The first took place between 90 and 200 °C and is associated with the elimination of EG and remaining functional groups on the rGO, and the second extended from 200 to 600 °C and is attributed to the degradation of the rGO carbon skeleton. The last event is catalyzed by the embedded Ni nanoparticles, as evidenced by the shift in the decomposition temperature from 565 to 325 °C (Figure 1D). The rGO/Ni/PVP-4 nanocomposite was also evaluated to check the influence of PVP. Besides the first mass loss event with the same attribution as rGO/Ni-4, there are some additional peaks in the derivative TGA (DTGA) at 267, 408, and 503 °C, which could be related to the degradation of PVP in addition to rGO. This higher degradation temperature is probably a result of the coating of the Ni NPs by PVP, which reduces the oxidative catalysis of rGO, thus increasing its stability. Moreover, the residue decreases from 61.3 to 55.9% when using PVP as an extra stabilizer, as expected due to the addition of a new carbon-based component. The temperature ranges, mass loss percentages, and average temperatures of the events are presented in Table 1.

**Table 1:** TGA Data of Samples in Air Atmosphere^a^

	1st event			2nd event			3rd event			
sample	TR	ET	ML%	TR	ET	ML%	TR	ET	ML%	*R*%
rGO/Ni-4	90–200	153	3.0	200–600	325	34.3				61.3
rGO/Ni/PVP-4	80–196	144	2.9	196–552	267/408/503	41.1				55.9
rGO	77–187	126	9.3	198–384	330	16.2	415–636	565	69.1	2.2

a TR: temperature range (°C); ET: event temperature (°C), from DTGA; ML%: mass loss %; *R*%: residue %.

The morphological and elemental characterization of the rGO/Ni nanocomposites were performed by SEM and EDS. The SEM images showed that the Ni nanoparticles were uniformly distributed across the rGO layers, with typical nanoparticle sizes ranging from 10 to 30 nm. As shown in Figure 2, the higher the reducing agent ratio, the smaller the metallic nanoparticle size, corroborating the XRD data. Smaller nanoparticles are desirable since a larger surface area plays a crucial role in improving the efficiency of glucose oxidation. However, analyzing low-magnification images (Figure S1 in the Supporting Information) shows that all samples are formed by a large quantity of Ni NPs, also producing agglomerates of NPs. The EDS spectra (Figure 2F) confirmed the expected elemental composition of the materials. All samples exhibited notable Ni and C signals, indicating the presence of metallic Ni and rGO, respectively. Remarkably, the rGO/Ni/PVP-4 nanocomposite exhibited a relatively stronger oxygen signal compared to rGO/Ni-4, which can be attributed to residual oxygen-containing groups on both rGO and PVP polymer. A weak nitrogen signal was detected in rGO/Ni/PVP-4, which served as a spectroscopic fingerprint of the pyrrolidone ring in PVP, and confirmed its incorporation. The C signal is related to rGO and, for rGO/Ni/PVP-4, also to PVP. These data evidence the presence of PVP on the rGO/Ni/PVP-4 sample. In addition, a small Na signal is present in the rGO/Ni/PVP-4 spectrum, probably due to the use of NaBH_4_. EDS mapping images were recorded for the rGO/Ni/PVP-4 nanocomposite (Figure S2), and these images show a distribution of Ni nanoparticles across the entire surface of the rGO, as well as C and O signals from both the rGO and PVP. The Ni:C mass ratio was calculated for all nanocomposites based on the elemental percentage of EDS spectra from 10 different regions per sample with a field of view of 210 μm. The results presented in Figure S3 show that the nanocomposites without PVP have a similar C:Ni ratio, considering the standard deviation. The rGO/Ni/PVP-4 nanocomposite, however, has a higher carbon content than the other samples, as verified in the TGA analyses.

**Figure 2:**
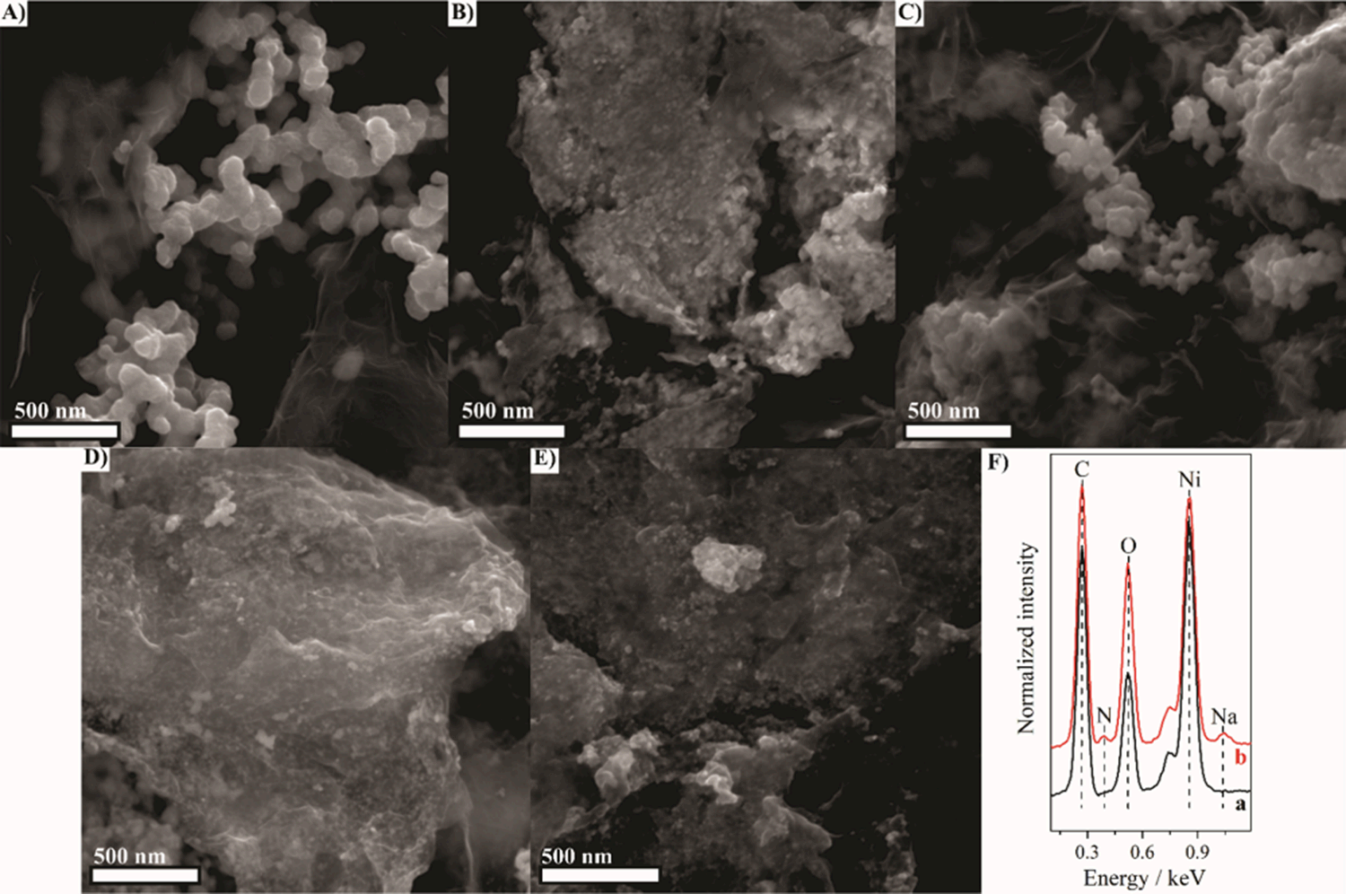
SEM images of (A) rGO/Ni-1, (B) rGO/Ni-2, (C) rGO/Ni-3, (D) rGO/Ni-4, and (E) rGO/Ni/PVP-4. (F) EDS spectra of rGO/Ni-4 (a), and rGO/Ni/PVP-4 (b).

TEM was used to further analyze the morphology, dispersion, and crystallinity of the Ni nanoparticles embedded in the rGO matrix. Representative bright-field and dark-field TEM images for rGO/Ni-3, rGO/Ni-4, and rGO/Ni/PVP-4 are shown in Figure 3. In all samples, the Ni nanoparticles are visible as high-contrast, spherical features that are uniformly anchored on the rGO nanosheets. In particular, the dark field images (Figure 3C,F,I), the Ni nanoparticles highlight the crystalline nature of the metallic Ni domains, which appear as bright diffraction spots distributed across the carbonaceous matrix. Histograms generated from the manual measurement of over 300 nanoparticles per sample showed a clear dependence of particle size on NaBH_4_/Ni^2+^ molar ratio. The average sizes progressively decreased with increasing reducing agent concentration: 9.5 ± 7.4 nm, for rGO/Ni-3, 4.0 ± 0.9 nm, for rGO/Ni-4, and 4.4 ± 1.0 nm, for rGO/Ni/PVP-4.

**Figure 3:**
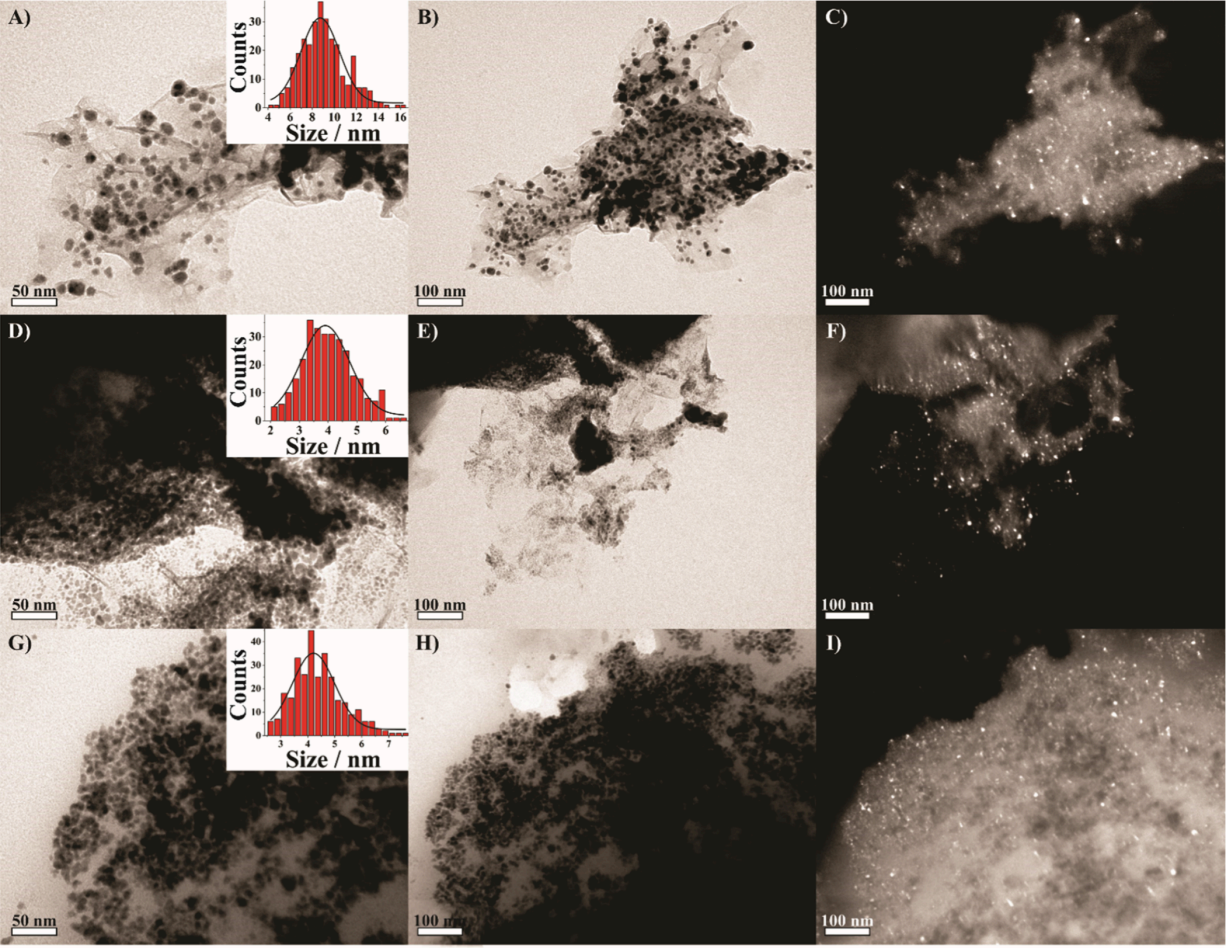
TEM images of (A–C) rGO/Ni-3, (D–F) rGO/Ni-4, and (G–I) rGO/Ni/PVP-4. Bright field images are shown in (A, B, D, E, G, H), while dark field images are shown in (C, F, I). The dark field images (C, F, I) correspond respectively to the same regions as (B, E, H).

### 2.2 Electrochemical Characterization and Electroanalytical Application

The electrochemical properties of the rGO/Ni nanocomposites were evaluated by cyclic voltammetry (CV) in 1 mol L^–1^ KOH. Representative voltammograms from the 50th scan are shown in Figure 4A. All samples exhibited a pair of well-defined redox peaks, corresponding to the electrochemically reversible conversion between Ni­(OH)_2_ and NiOOH, and indicating that the metallic Ni nanoparticles were converted to Ni­(OH)_2_ during CV. A systematic increase in peak current with decreasing nanoparticle size was observed, emphasizing the crucial role of the surface area and density of the electroactive sites. Among all nanocomposites, the rGO/Ni/PVP-4 sample showed the highest redox response, with anodic peak current of more than ∼140 μA cm^–2^, which was significantly higher than those of the other compositions. This superior behavior is attributed to the synergistic effect of PVP, which improves nanoparticle dispersion, reduces aggregation, and stabilizes smaller particles with a larger active surface area, facilitating the conversion of Ni to Ni­(OH)_2_, as well as in the Ni­(OH)_2_/NiOOH redox reaction (Figure 5). The electroactive surface area of the ITO-modified electrodes, after pretreatment by 50 CV cycles in 1 mol L^–1^ KOH, was evaluated using potassium ferrocyanide as an electrochemical probe in 0.1 mol L^–1^ NaCl (Figure S4). By applying different scan rates (5–100 mV s^–1^), and the Randles–Sevcik equation, the electrochemically active area was determined (Figure S4F).^8,19^ The results indicate that increasing the molar ratio of reducing agent led to a slight increase in the electroactive area, suggesting that the smaller size of the Ni nanoparticles contributed to a higher density of electroactive sites. In addition, the interfacial interaction between Ni and rGO facilitates rapid electron transport across the interface, and maximizes the electrochemical response. These results confirm that the combination of nanostructuring, surface chemistry, and polymer stabilization plays a crucial role in improving the redox performance of the nanocomposite platform.

**Figure 4:**
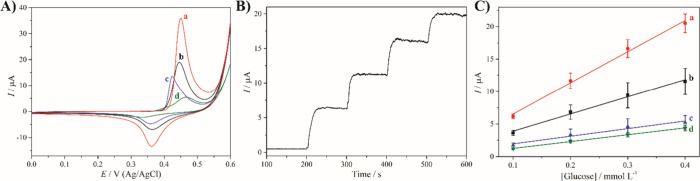
(A) 50th cyclic voltammograms, (B) chronoamperogram, and (C) analytical curves of rGO/Ni/PVP-4 (a), rGO/Ni-4 (b), rGO/Ni-3 (c), and rGO/Ni-2 (d). The chronoamperogram in (B) corresponds to the rGO/Ni/PVP-4 nanocomposite. The electrochemical measurements were carried out in 1 mol L^–1^ KOH, and the cyclic voltammograms were recorded at 50 mV s^–1^. The chronoamperograms were recorded by applying the anodic peak potential under magnetic stirring with successive additions of 0.1 mmol L^–1^ glucose after voltammetric pretreatment.

**Figure 5:**
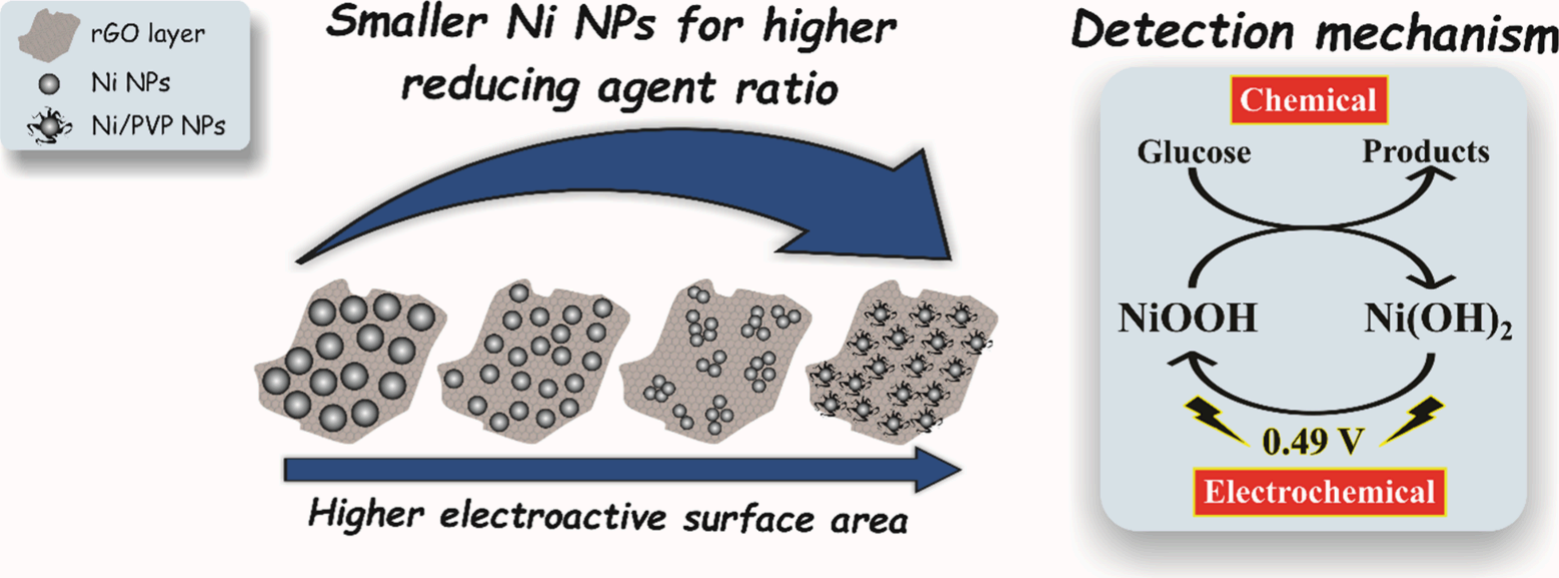
Schematic representation of the influence of the Ni:NaBH_4_ molar ratio on the size of Ni NPs in nanocomposites, as well as the presence of PVP as a stabilizer, and glucose detection mechanisms.

The electroanalytical performance of the rGO/Ni nanocomposites for the detection of glucose was evaluated by chronoamperometry (CA) in 1 mol L^–1^ KOH, using the anodic peak potential previously determined by CV. As shown in Figure 4B, successive addition of 0.1 mmol L^–1^ glucose resulted in a stepwise increase in current, confirming the electrocatalytic activity of the nanocomposites for glucose oxidation. The recognition mechanism involves the chemical oxidation of glucose by NiOOH, which is continuously regenerated from Ni­(OH)_2_ under the applied potential (Figure 5). This redox cycle maintains a stable catalytic current, that increases in proportion to the glucose concentration, as previously described for Ni-based materials in alkaline media.^8^ Analytical calibration curves were constructed based on the current increase after each addition (Figure 4C). The rGO/Ni/PVP-4 thin film exhibited the highest sensitivity, which was calculated to be 48.6 ± 2.8 μA (mmol L^–1^)^−1^, significantly outperforming the other nanocomposites: 26.4 ± 5.6 μA (mmol L^–1^)^−1^ for rGO/Ni-4, 11.7 ± 2.5 μA (mmol L^–1^)^−1^ for rGO/Ni-3, and 10.5 ± 1.0 μA (mmol L^–1^)^−1^ for rGO/Ni-2. These results emphasize the key role of the size and dispersion of the nanoparticles for the sensing properties (Figure 5), since smaller sizes increase the surface area and consequently the electroactive area of the nanocomposite. The improved performance of the PVP-containing film is attributed to the increased electroactive surface area, improved redox cycling, and better mass transfer conditions. Of all the materials studied, rGO/Ni/PVP-4 not only provided the highest catalytic current, but also showed the most consistent and linear response to the addition of glucose.

The electrocatalytic response of the rGO/Ni/PVP-4 nanocomposite was optimized by conducting several experiments varying the applied potential in the chronoamperometry (Figure 6). The analytical curves demonstrate a relationship between sensitivity and the applied potential for glucose detection. The higher the applied potential, the higher the sensitivity. This behavior is expected, since a higher potential favors the formation of the catalytic species (NiOOH). However, sensitivity decreases when 0.520 V is applied. This is likely due to the oxidation of water by the nanocomposite, which competes with the oxidation of glucose and leads to gas evolution, thus decreasing the contact of the analyte with NiOOH.^8^ Therefore, the optimized potential for chronoamperometric glucose detection was 0.49 V for the rGO/Ni/PVP-4 thin film.

**Figure 6:**
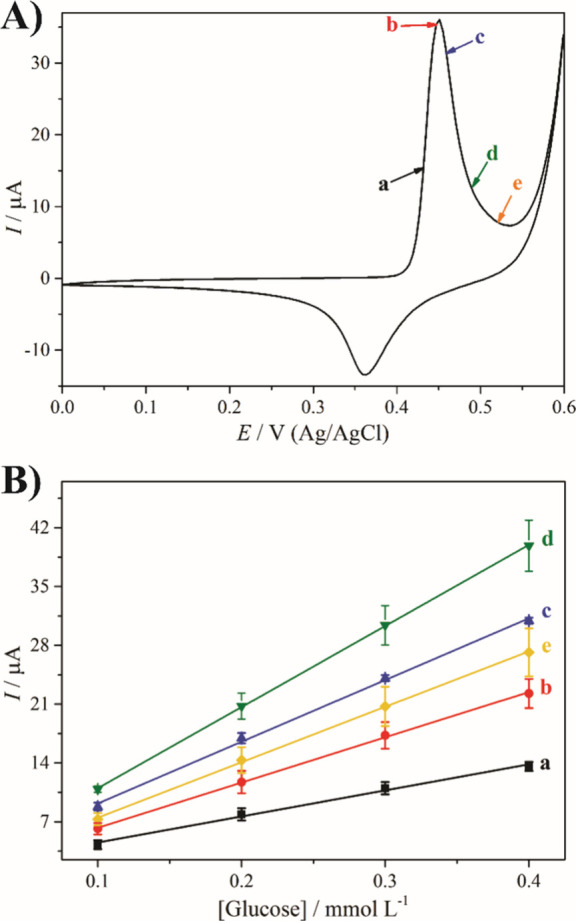
(A) 50th cyclic voltammogram and (B) analytical curves of rGO/Ni/PVP-4 applying 0.430 (a), 0.445 (b), 0.460 (c), 0.490 (d), and 0.520 V (e). The electrochemical measurements were carried out in 1 mol L^–1^ KOH, and the cyclic voltammograms were recorded at 50 mV s^–1^. The chronoamperograms were recorded under magnetic stirring with successive additions of 0.1 mmol L^–1^ glucose, after voltammetric pretreatment.

Glucose detection was further carried out over a wide concentration range, and the chronoamperogram as well as the analytical curve are shown in Figure 7. The rGO/Ni/PVP-4 nanocomposite exhibited electroactivity throughout the entire glucose concentration range (from 1 to 1000 μmol L^–1^). Considering the sensitivity for the initial glucose additions (64.0 ± 6.0 μA (mmol L^–1^)^−1^), the limit of detection (LOD), and the limit of quantification (LOQ) were calculated using eqs 1 and 2, respectively:^18^

$$\mathrm{LOD}=\frac{3s}{\alpha }$$ 1

$$\mathrm{LOQ}=\frac{10s}{\alpha }$$ 2

where *s* is the standard deviation (0.006 μA) from the blank, and α is the sensitivity. The optimized rGO/Ni/PVP-4 thin film exhibited very low values of LOD, and LOQ, reaching 0.29 ± 0.06, and 0.95 ± 0.18 μmol L^–1^, respectively. To further evaluate the performance of the rGO/Ni/PVP-4 sensor, its analytical properties were compared with those of other nonenzymatic glucose sensors reported in the literature (Table 2). While several reported systems show an extended detection range, many of them suffer from lower sensitivity or higher LOD. For example, Pt/MXen-based sensors show wide linear ranges but relatively poor sensitivity (0.21 μA mmol L^–1^)^−1^) and high LODs (>29 μmol L^–1^).^20^ Similarly, some metal–organic frameworks (MOFs) offer extremely high sensitivity, but in narrower detection windows or under very specific conditions.^21,22^ In contrast, the rGO/Ni/PVP-4 sensor showed a balanced combination of important analytical parameters: a wide linear detection range (1–1000 μmol L^–1^), high sensitivity (64.0 ± 6.0 μA (mmol L^–1^)^−1^), and remarkably low LOD (0.29 ± 0.06 μmol L^–1^). With this performance, the developed nanocomposite sensor is one of the most competitive platforms for nonenzymatic glucose detection. The success lies in the optimized synthesis protocol, which results in small, well-dispersed and PVP-stabilized Ni nanoparticles that are tightly bound to the conductive rGO matrix. These properties improve the redox cycle and surface adsorption, both of which are crucial for fast and sensitive detection.

**Figure 7:**
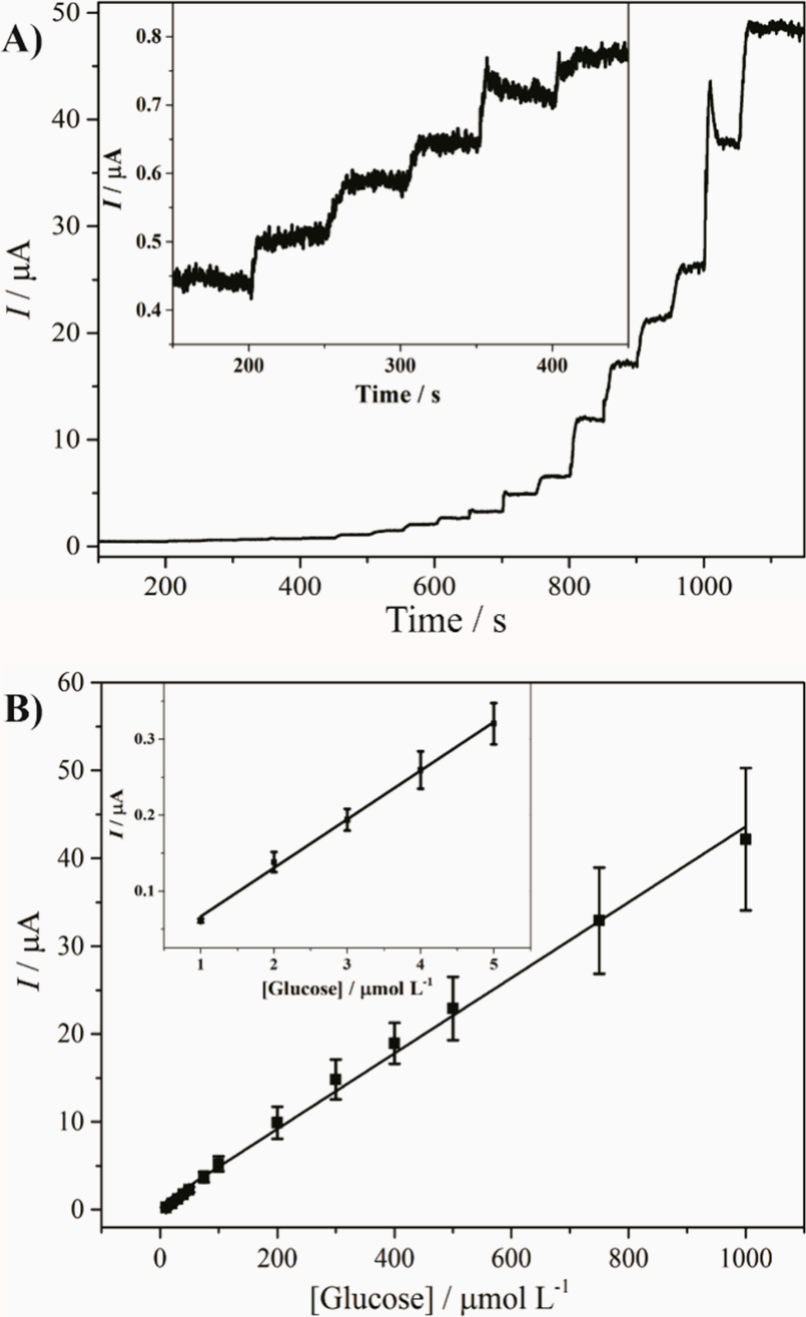
(A) Chronoamperogram, and (B) analytical curve of rGO/Ni/PVP-4 applying 0.49 V and using a glucose concentration range from 1 to 1000 μmol L^–1^. The insets in (A), and (B) correspond to an enlargement of the first five glucose additions.

**Table 2:** Comparison of the Optimized rGO/Ni/PVP-4 Thin Film Sensor with Previously Published Reports

sensor	method	linear detection range (μmol L^–1^)	sensitivity (μA (mmol L^–1^)^−1^)	LOD (μmol L^–1^)	ref
rGO/Ni(OH)_2_	chronoamperometry	1–1000	64.0 ± 6.0	0.29 ± 0.06	this work
SNF/rGO/Gox^a^	chronoamperometry	0–100	18	0.3	^23^
Pt/MXene^b^	chronoamperometry	0–8000	0.21	29.15	^20^
NiCo-MOF/GO^c^	chronoamperometry	1–497	790.09	0.23	^21^
Ni-MOF^d^	chronoamperometry	1–1600	7.52 × 10^–4^	0.76	^22^
Cu_*x*_O/NPC@Co_3_O_4_/NPC-10-7^e^	chronoamperometry	2.5–1078	2.32 × 10^–4^	0.62	^24^

a Silk nanofibril with rGO and glucose oxidase.

b MXene (Ti_3_C_2_T_*x*_) decorated with Pt nanoparticles.

c Ni-Co based metal organic frameworks (MOF) attached to graphene oxide.

d MOF decorated with Ni nanoparticles.

e N-Doped porous carbon-supported Cu/Co oxides by high-temperature pyrolysis.

As can be seen in Figure 8A, the sensor showed excellent reproducibility across seven independently prepared electrodes, with sensitivity values showing a relative standard deviation (RSD) of 13.1%. In terms of repeatability, the same modified electrode was tested over four consecutive glucose additions under identical conditions. As can be seen in Figure 8B, a progressive decrease in sensitivity was observed with each subsequent measurement. The response dropped significantly after the second run, indicating partial deactivation or leaching of the active material from the electrode surface. This limited reusability can be attributed to the weak adhesion of the nanocomposite to the ITO substrate or to the mechanical stress during stirring. Despite this limitation, the high initial sensitivity and reproducibility make the rGO/Ni/PVP-4 thin film a promising candidate for single-use, sensing applications, where consistent performance and low production costs take precedence over long-term reusability.

**Figure 8:**
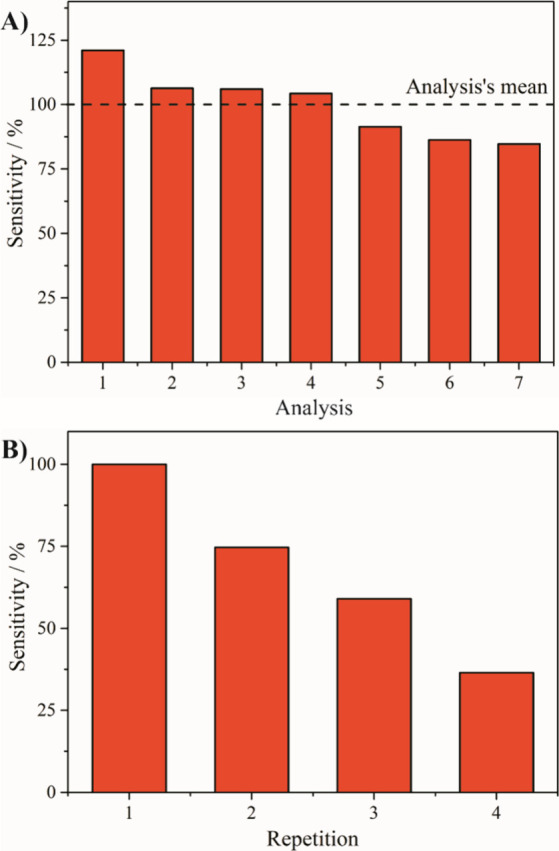
(A) Reproducibility, and (B) repeatability of the rGO/Ni/PVP-4 thin film for glucose detection in 0.1 mol L^–1^ KOH applying 0.49 V. The sensitivities in (A), and (B) are expressed as percentages of the mean sensitivity of all experiments, and of the sensitivity of the first experiment, respectively.

The selectivity of the rGO/Ni/PVP-4 sensor for glucose was evaluated by CA in the presence of common interferents typically found in biological or environmental samples, such as NaCl, KCl, UA, urea, AA and fructose.^25^ As can be seen in Figure 9, the addition of 0.1 mmol L^–1^ glucose resulted in a significant increase in current, confirming the electrocatalytic activity of the sensor. In contrast, the subsequent addition of 1 mmol L^–1^ NaCl, KCl, urea, and fructose resulted in negligible current responses, indicating minimal interference with the glucose oxidation process. The addition of 1 mmol L^–1^ UA, and AA resulted in a slight increase in current, which can be attributed to its known electroactivity at similar potentials. However, the sensitivities generated by UA, and AA were lower than to glucose reaction (52.3, 45.1, and 69.8 μA mmol L^–1^ for UA, AA, and glucose, respectively), indicating that the sensor retains a high degree of selectivity even in the presence of potentially interfering compounds. This selectivity can be explained by the specific redox interaction between NiOOH and glucose molecules, which involves a direct chemical oxidation pathway. In addition, the presence of PVP, and the high dispersion of Ni nanoparticles on the rGO surface likely contribute to minimize nonspecific adsorption, and improve the electrochemical discrimination of glucose over other analytes. After all the interferent additions, glucose was added again, but no signal was observed. The absence of a current increase suggests poisoning or saturation of the electroactive sites due to the previous interferent additions (a total of 6 mmol L^–1^).

**Figure 9:**
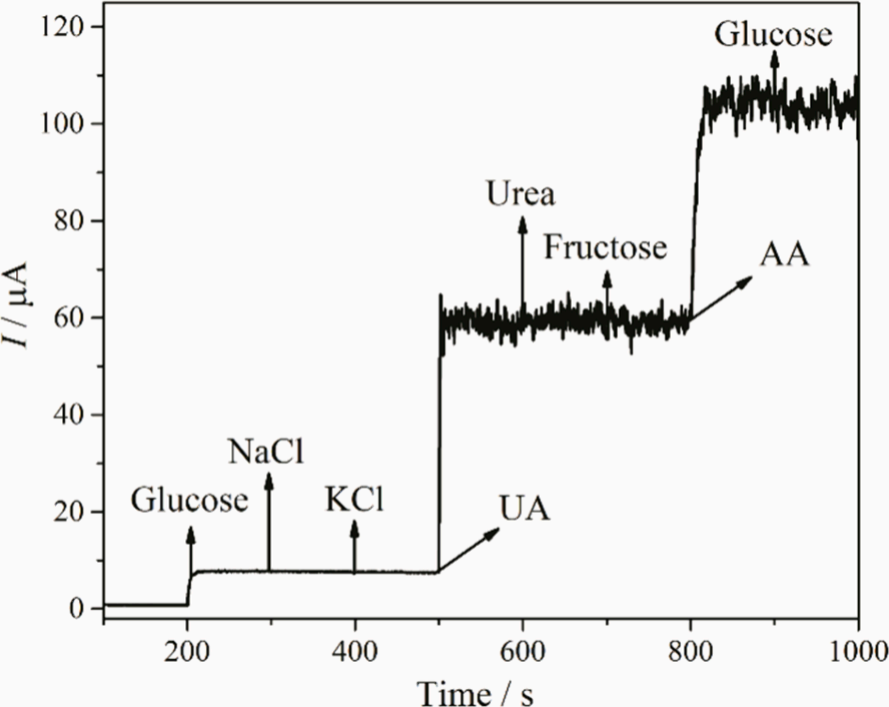
Chronoamperogram of the rGO/Ni/PVP-4 thin film with additions of 0.1 mmol L^–1^ glucose and 1 mmol L^–1^ NaCl, KCl, UA, urea, fructose, AA, and 0.1 mmol L^–1^ glucose.

## 3 Conclusions

In this study, a series of nickel-based nanocomposites with reduced graphene oxide (rGO/Ni) were synthesized using NaBH_4_ as a reducing agent, either in the presence or absence of PVP as a stabilizing agent. Physicochemical characterizations confirmed the successful formation of metallic Ni nanoparticles with controlled size and uniform distribution in the rGO matrix, by controlling the Ni^2+^:NaBH_4_ ratio. Among the synthesized materials, the rGO/Ni/PVP-4 nanocomposite exhibited the smallest particle size (∼4 nm), and improved structural homogeneity, leading to increased active site density. Electrochemical characterization in alkaline medium showed a clear redox reaction associated with the Ni­(OH)_2_/NiOOH pair, with narrow peak separation and stable current intensities. Chronoamperometric measurements showed that the rGO/Ni/PVP-4 thin film exhibited the highest sensitivity to glucose (64.0 ± 6.0 μA (mmol L^–1^)^−1^), a low LOD (0.29 ± 0.06 μmol L^–1^), and excellent reproducibility (RSD = 13.1% across all electrodes). Although the repeatability of the sensor decreased slightly over several applications—probably due to weak adhesion or partial loss of the active material—it remained highly selective for glucose, and showed minimal or no interference. These results highlight the potential of the rGO/Ni/PVP-4 sensor as a low-cost, nonenzymatic, and highly sensitive electrochemical platform for glucose detection, offering promising applications in disposable sensor technologies for biomedical or environmental analysis.

## 4 Materials and Methods

### 4.1 Reagents

Sodium nitrate (NaNO_3_, Vetec, analytical grade), Graphite (Graflake 99580, 99%, Nacional de Grafite SA), sulfuric acid (H_2_SO_4,_ Nuclear, 98.0%), potassium permanganate (KMnO_4_, Synth, analytical grade), hydrochloric acid (HCl, Dinâmica, 37%), hydrogen peroxide (H_2_O_2_, Synth, 30% V/V), sodium borohydride (NaBH_4_, Sigma-Aldrich, 99%), polyvinylpyrrolidone (PVP, Sigma-Aldrich, 95%), acetone (Quimidrol, >99.5%), ethylene glycol (EG, Vetec, 99.5%), glucose (Sigma-Aldrich, analytical grade), sodium chloride (NaCl, analytical grade, Vetec), potassium chloride (KCl, analytical grade, Dinâmica), uric acid (UA, 99%, Sigma), urea (99.5%, Merck), and fructose (99%, Sigma) were used as received. The nickel­(II) acetate tetrahydrate (Ni­(CH_3_COO)_2_·4H_2_O, Sigma-Aldrich, 98%) was dried at 100 °C for 5 h to remove the adsorbed water. The aqueous solutions and the washing steps were realized using deionized water.

### 4.2 Oxidation of Graphite and Obtaining the GO

For the oxidation of graphite, as previously performed by our research group,^18^ 2 g of graphite was combined with 1 g of NaNO_3_ and transferred to a 500 mL round-bottom flask, which was placed in an ice bath to keep the temperature low during the addition of the reagents. Next, 46 mL of concentrated H_2_SO_4_ was gradually added to the mixture and magnetically stirred at approximately 1500 rpm for 45 min to ensure uniform agitation. Then, 6 g of KMnO_4_ was slowly added to the system. Once the KMnO_4_ was fully incorporated, the ice bath was removed and the reaction continued at room temperature for approximately 3 h.

The mixture was carefully treated with 92 mL of water, followed by another 280 mL of hot water (approximately 90 °C) to ensure a controlled temperature rise and minimize side reactions. Subsequently, 10 mL of hydrogen peroxide was slowly added to neutralize the remaining oxidants in the system. After 30 min of magnetic stirring, the reaction mixture was transferred to a beaker and treated with 500 mL of a 3.7% hydrochloric acid aqueous solution to remove sulfate ions. The reaction mixture was then stirred for a further 10 min and allowed to settle to allow solid particles to precipitate. The precipitated solid was washed several times with deionized water until the supernatant reached a neutral pH of about 7.00.

The purified solid, now consisting mainly of graphite oxide (Gr-O), was filtered and dried at 60 °C for 24 h to remove residual moisture. To further exfoliate the Gr-O flakes, 75 mg of Gr-O was dispersed in 25 mL of deionized water (3 mg mL^–1^ concentration) and subjected to ultrasonic treatment in an ultrasonic bath (Ultronique, 37 kHz) for 60 min. The mixture was then centrifuged at 3000 rpm for 30 min to separate the nonexfoliated material. The supernatant containing the exfoliated graphene oxide (GO) was then collected and subjected to a second centrifugation under the same conditions to ensure maximum separation efficiency.

The final GO dispersion was concentrated by heating on a hot plate at 80 °C with constant magnetic stirring. After concentration, the dispersion was transferred to an oven and dried at 60 °C for 3 days. This drying process resulted in a fine, powdered form of GO ready for subsequent applications such as the synthesis of nanocomposites.

### 4.3 Synthesis of the rGO/Ni Nanocomposites

The rGO/Ni nanocomposites were synthesized using a modified polyol method aimed at optimizing the size and distribution of nickel nanoparticles on the reduced graphene oxide (rGO) support.^17^ Initially, 5 mg of GO was dispersed in 20 mL of EG in a 250 mL round-bottom flask. The dispersion was sonicated in an ultrasonic bath for 60 min to ensure uniform exfoliation of the GO layers. After sonication, 22.1 mg of nickel acetate tetrahydrate was added to the flask, which was then fitted into a reflux system under continuous magnetic stirring at 1500 rpm. Once the temperature of the reaction mixture reached 180 °C, sodium borohydride—used as a reducing agent—was rapidly introduced into the system. The reduction process was maintained for 2 h, after which the reaction was cooled to room temperature spontaneously, and the solid product was precipitated by adding acetone. The mixture was then centrifuged at 4500 rpm for 20 min to collect the solid. This washing and centrifugation step was repeated three times to ensure the complete removal of unreacted reagents and byproducts. The purified solid was subsequently dried at 100 °C for 2 h.

A series of rGO/Ni nanocomposites with different Ni^2+^:NaBH_4_ molar ratios were synthesized. Specifically, 4.5, 13.5, 27, or 54 mg of NaBH_4_ were used to obtain 1:1 (rGO/Ni-1), 1:3 (rGO/Ni-2), 1:6 (rGO/Ni-3), and 1:12 (rGO/Ni-4) molar ratios, respectively. Additionally, a rGO/Ni nanocomposite was synthesized using PVP as an extra stabilizing agent, based on the 1:12 Ni^2+^:NaBH_4_ molar ratio (rGO/Ni/PVP-4). In this case, after the addition of nickel acetate, 44 mg of PVP was introduced into the system, resulting in a Ni^2+^:PVP mass ratio of 1:6.

### 4.4 Characterizations

X-ray diffraction (XRD) analyses were performed using a Shimadzu XRD-6000 instrument with CuKα radiation (λ = 1.5418 Å) operated at 30 mA and 40 kV.

Fourier-transform infrared (FTIR) spectra were obtained with a Bruker instrument in attenuated total reflection (ATR) mode, using a resolution of 4 cm^–1^ and 20 scans per sample. A baseline correction was performed for the spectra of all nanocomposites.

Thermogravimetric analysis (TGA) was conducted on a PerkinElmer 4000 under synthetic air atmosphere, with a temperature range from 60 to 800 °C at a heating rate of 10 °C min^–1^. Samples were pretreated at 100 °C for 20 min to remove adsorbed moisture.

Scanning electron microscopy (SEM) images were acquired on a Tescan Field Emission Gun (SEM-FEG) system operating at 10 kV with a secondary electron detector. Samples were prepared by depositing powders on copper conductive tapes. Energy-dispersive X-ray spectroscopy (EDS) was performed using the same instrument equipped with an Oxford detector.

Transmission electron microscopy (TEM) images were recorded using a Jeol JEM microscope operating at 120 kV in both bright and dark field modes. For TEM preparation, nanocomposites were dispersed in water via ultrasonic treatment and then drop-cast onto carbon ultrathin films supported on copper grids, followed by drying at room temperature.

Electrochemical measurements were performed using a Palmsens potentiostat in a three-electrode configuration. Modified glass substrates coated with indium tin oxide (ITO) served as the working electrode (WE), an Ag/AgCl (saturated NaCl) electrode was the reference electrode (RE), and a platinum wire acted as the auxiliary electrode (AE). All measurements were carried out in 1 mol L^–1^ KOH electrolyte. Cyclic voltammograms of the thin films were recorded from 0.1 to 0.6 V at a scan rate of 50 mV s^–1^. Chronoamperometric glucose detection was conducted by applying the anodic peak potential or other specified potentials under magnetic stirring. Reproducibility and repeatability were assessed by performing four successive additions of 0.1 mmol L^–1^ glucose. The electroactive surface area of the modified ITO electrodes was evaluated by cyclic voltammetry in 0.1 mol L^–1^ NaCl containing 1 mmol L^–1^ K_4_[Fe­(CN)_6_], after pretreatment with 50 voltammetric cycles in 1 mol L^–1^ KOH.

The 0.5 × 2.5 × 0.11 cm ITO substrates (WellJoin, resistance <20 Ω □^–1^) were modified by delimiting an active area of 0.25 cm^2^ using parafilm, onto which 20 μL of a 0.1 mg mL^–1^ aqueous dispersion of the nanocomposites was drop-cast. The modified ITO electrodes were dried at 60 °C for 1 h, after which the parafilm was removed. Finally, electrodes were dried at 100 °C for 1 h to ensure good adhesion of the nanocomposite. The nanocomposite dispersion in water was prepared by ultrasonic treatment for 1 h.

## Supplementary Material



## Notes

SEM images, EDS mapping, C:Ni mass ratio from EDS spectra, and cyclic voltammograms in 0.1 mol L^–1^ NaCl solution containing 1 mmol L^–1^ of K_4_[Fe­(CN)_6_] of the nanocomposites (PDF)
