# Supplementary material for: Reduced Graphene Oxide and Nickel Nanoparticle Nanocomposites: Tailoring the Nanoparticle Size for Enhanced Glucose Electrochemical Sensors
Source: ACS Omega. 2026 May 4;11(19):28641–51. doi: 10.1021/acsomega.6c01043 (PMC13191518; doi:10.1021/acsomega.6c01043)
Supplement: Supplementary file 1 [file ao6c01043_si_001.pdf]

Reduced graphene oxide and nickel nanoparticles nanocomposites: tailoring the nanoparticles size for enhanced glucose electrochemical sensors

Anna E. Silva[1], Lízia A. X. Bulin[2], Jean C. Bassani[2], Drochss Valencia[3,4], Daniela Z. Mezalira[2] and Eduardo G.C. Neiva[1*]

1- *Departamento de Química, Universidade Regional de Blumenau (FURB), CEP 89012900, Blumenau, SC, Brazil.*
2- *Departamento de Química, Universidade Federal de Santa Catarina (UFSC), CEP 88040900, Florianópolis, SC, Brazil.*
3- *Escuela de Química, Universidad Industrial de Santander, Z.P. 680002, Bucaramanga, Santander, Colombia.*
4- *Servicio Geológico Colombiano, Dirección de Laboratorios, Z.P. 111321, Bogotá, Colombia.*

Supplementary material

* Corresponding author

Phone: +55-47-3321-0541

E-mail address: eneiva@furb.br

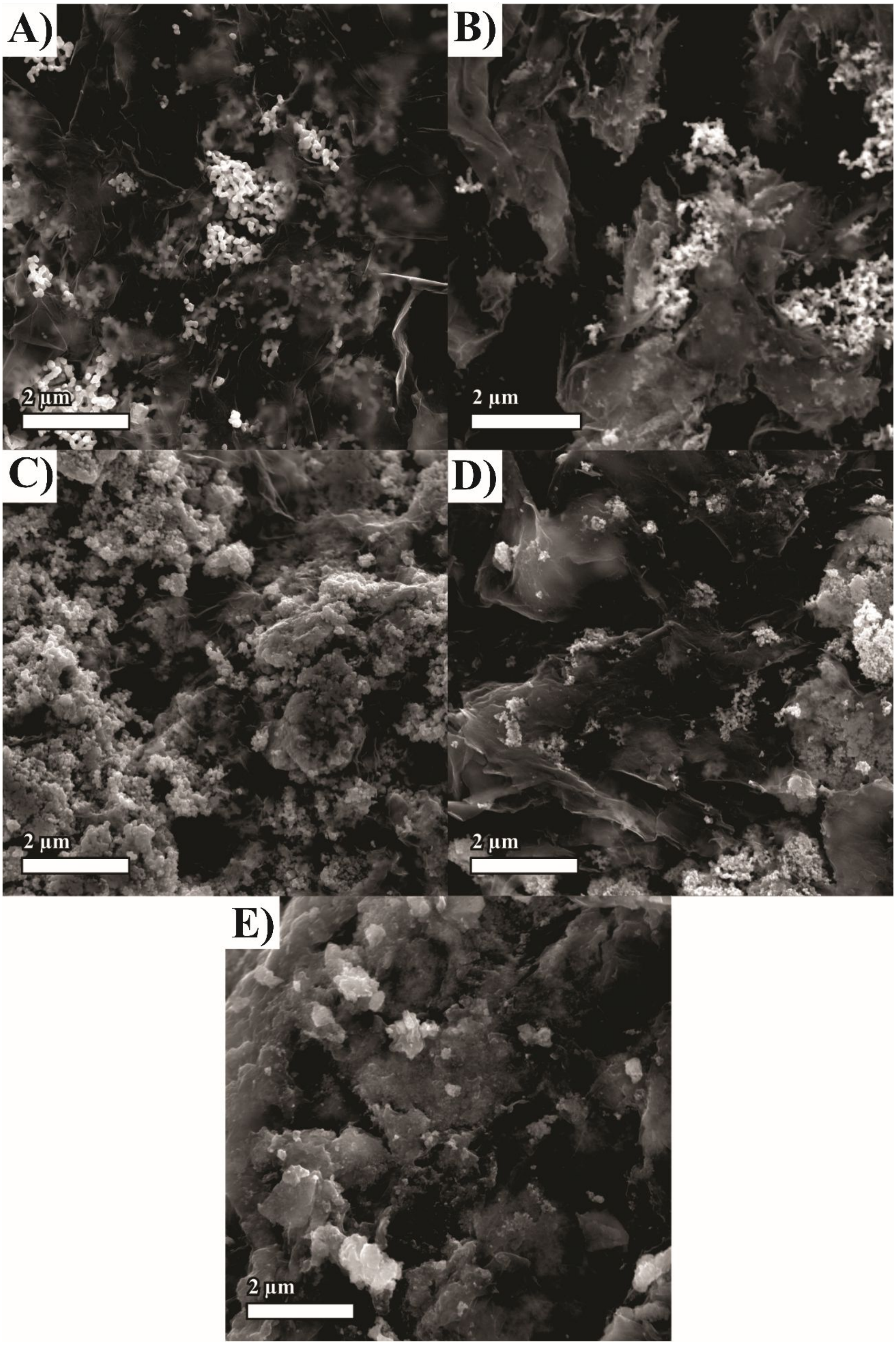

Figure S1. SEM images of (A) rGO/Ni-1, (B) rGO/Ni-2, (C) rGO/Ni-3, (D) rGO/Ni-4, and (E) rGO/Ni/PVP-4.

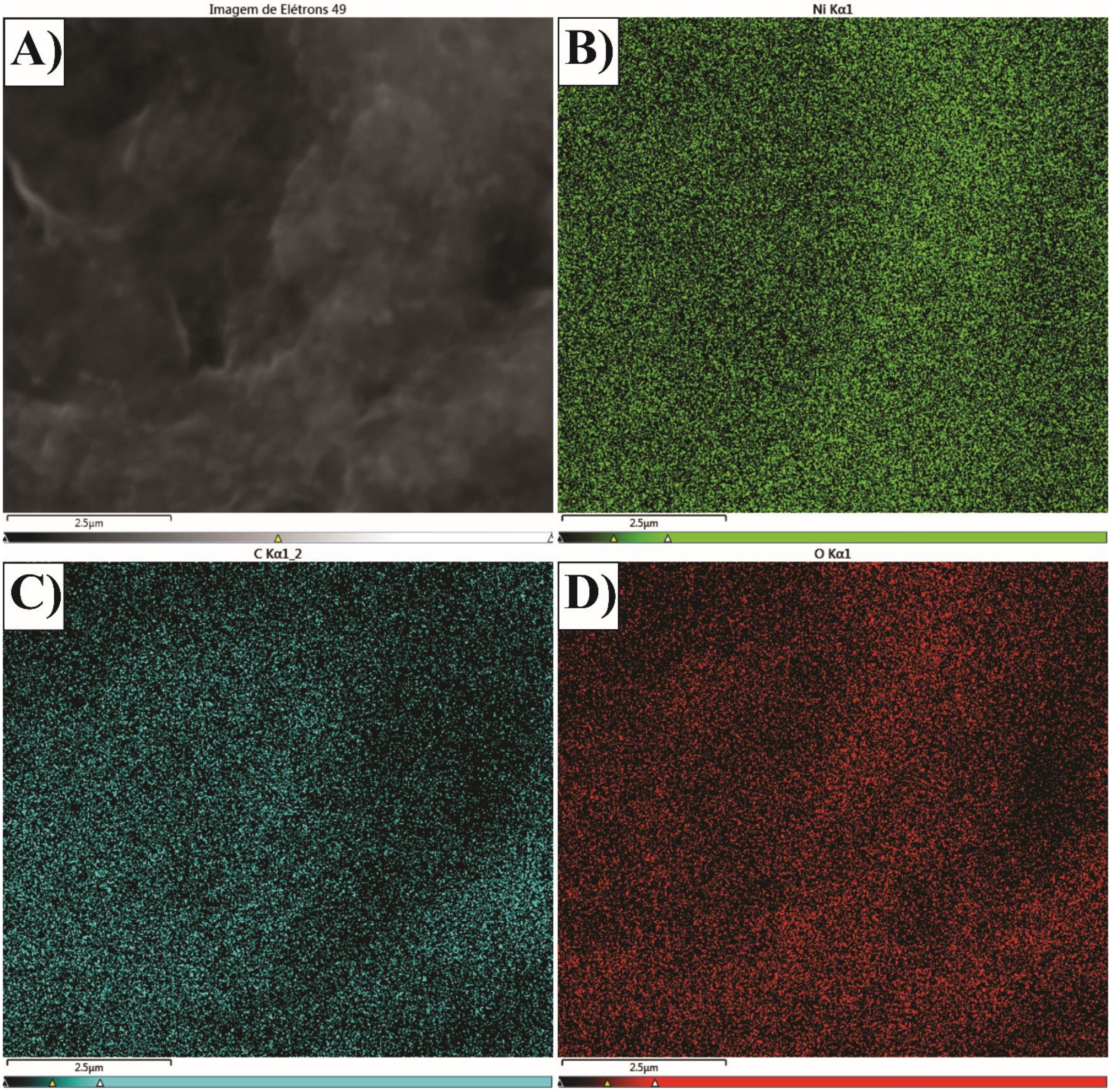

Figure S2. SEM image (A) and EDS mapping of Ni (B), C (C), and O (D) for rGO/Ni/PVP-4.

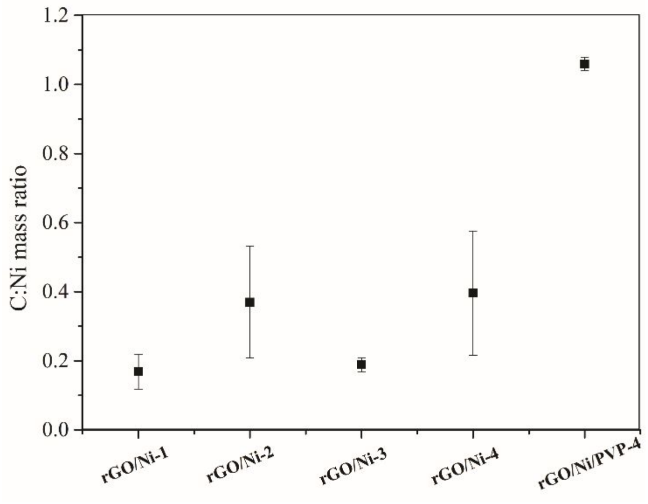

Figure S3. C:Ni mass ratio for rGO/Ni-1, rGO/Ni-2, rGO/Ni-3, rGO/Ni-4, and rGO/Ni/PVP-4 determined from *wt%* values obtained from EDS spectra. For each sample, ten spectra were acquired from regions with a field of view of 210 µm.

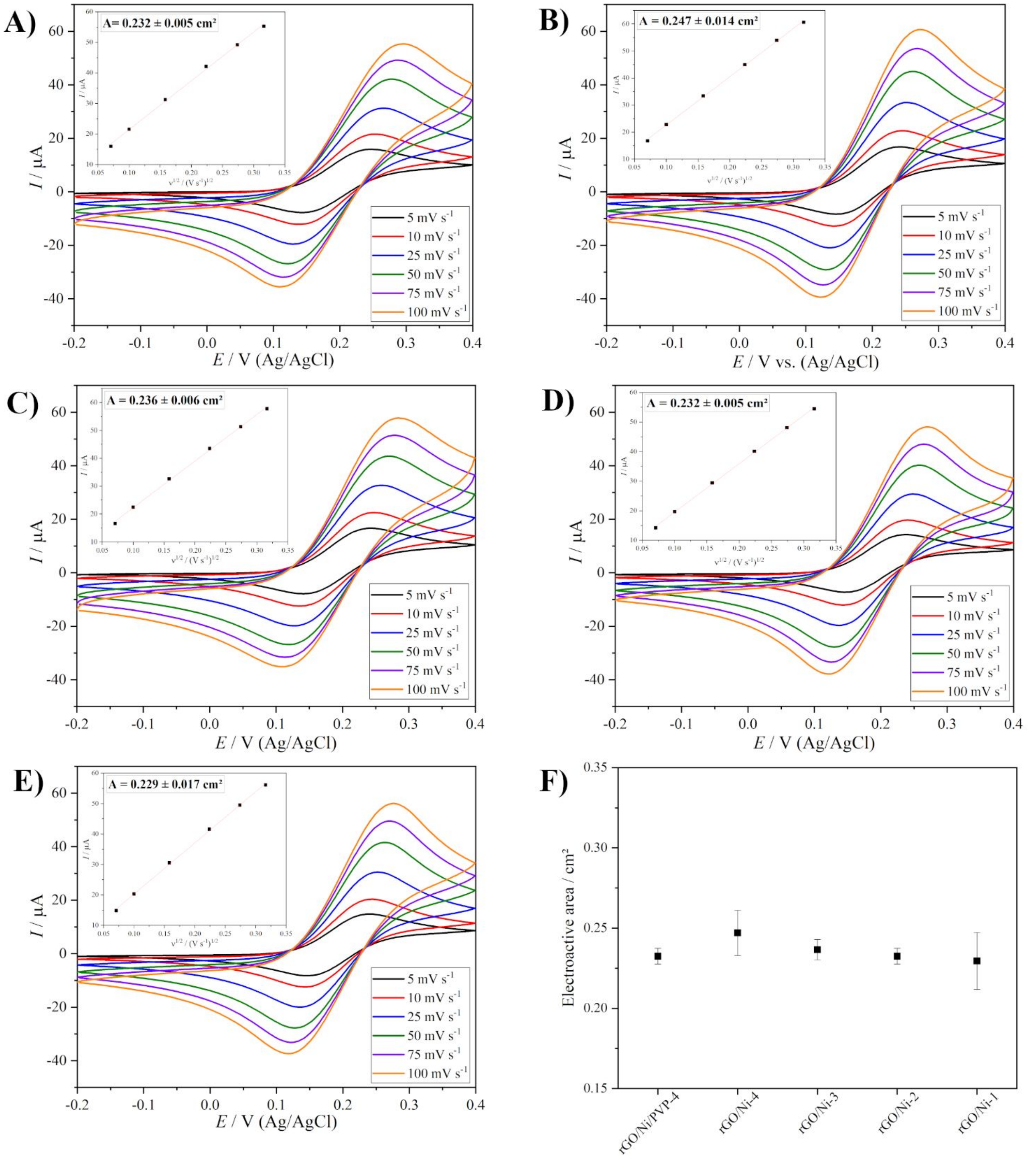

Figure S4. Cyclic voltammograms of ITO electrodes modified with (A) rGO/Ni/PVP-4, (B) rGO/Ni-4, (C) rGO/Ni-3, (D) rGO/Ni-2, and (E) rGO/Ni-1 in 0.1 mol L^{-1} NaCl solution containing 1 mmol L^{-1} of $K_4[Fe(CN)_6]$. The insets show plots of anodic peak current *vs.* square root of scan rate ($v^{1/2}$). (F) Comparison of the electroactive areas of the modified electrodes.
